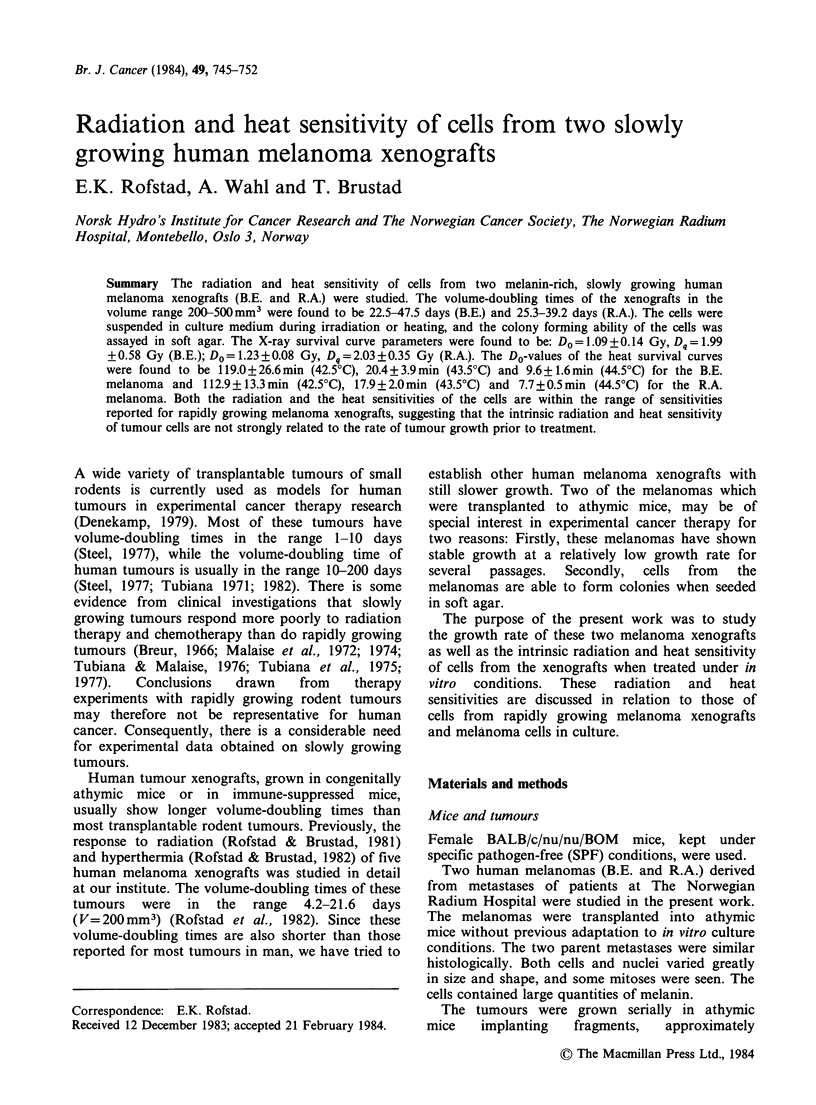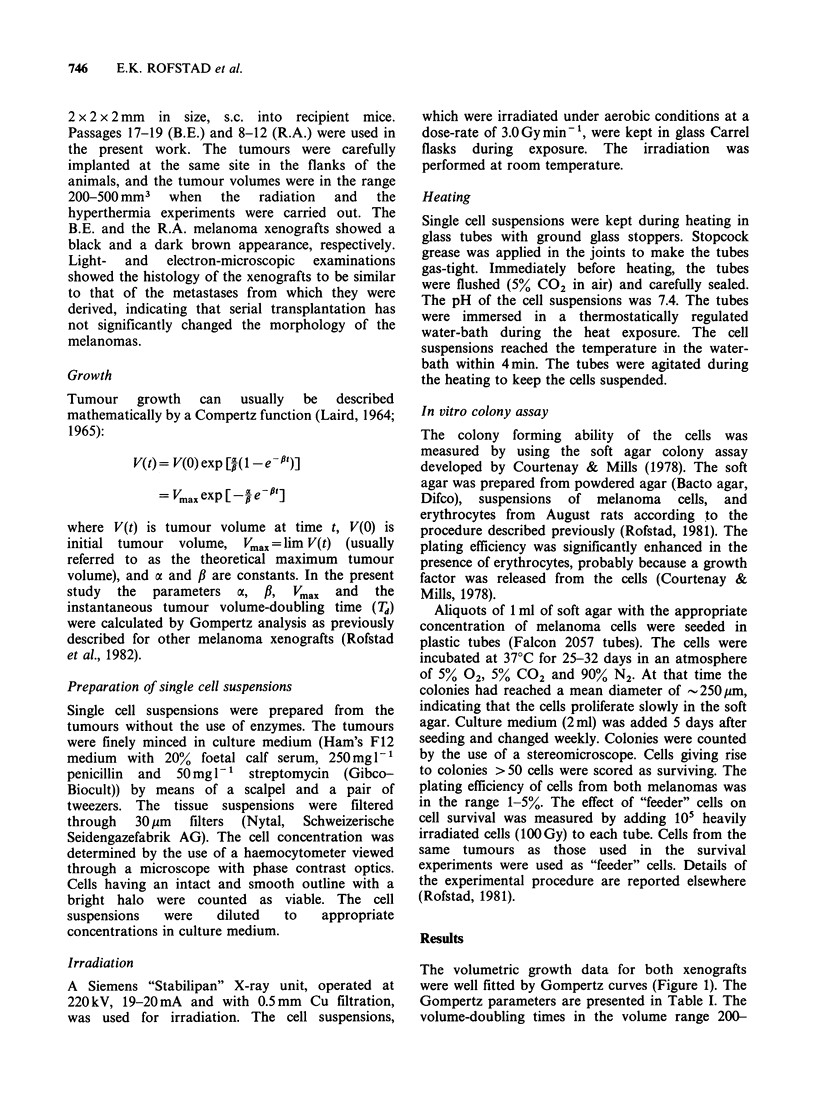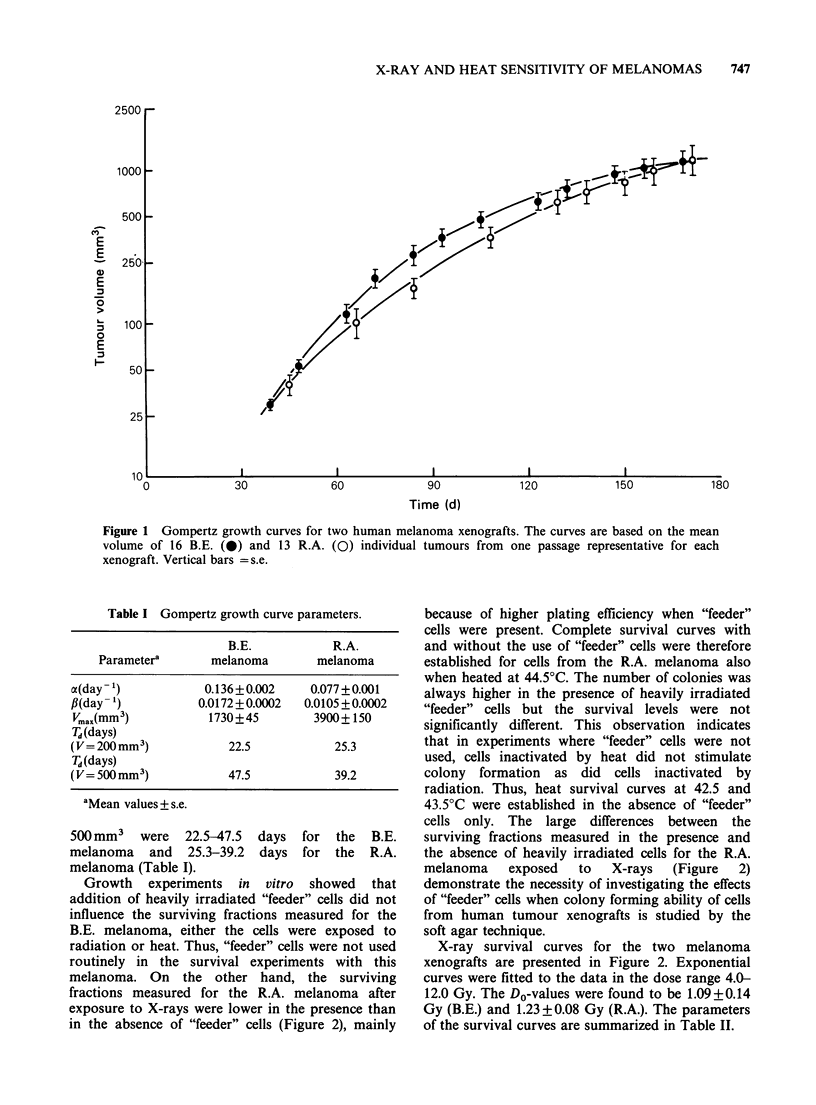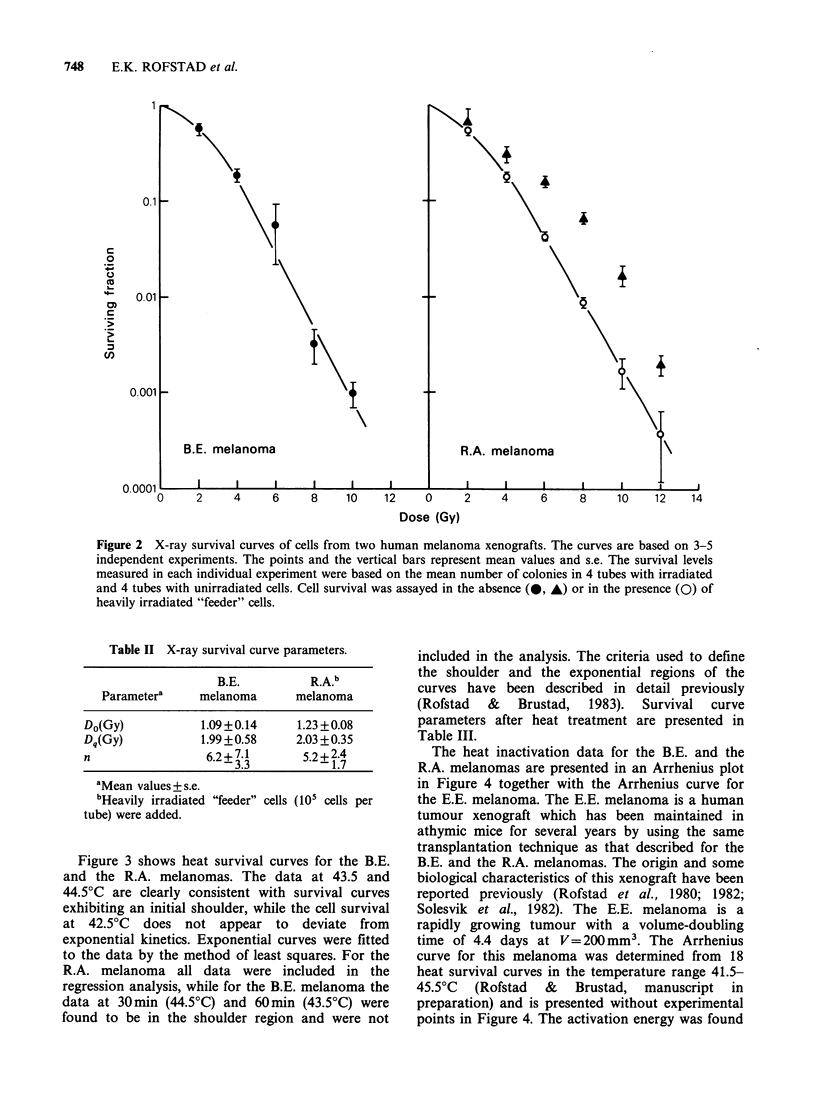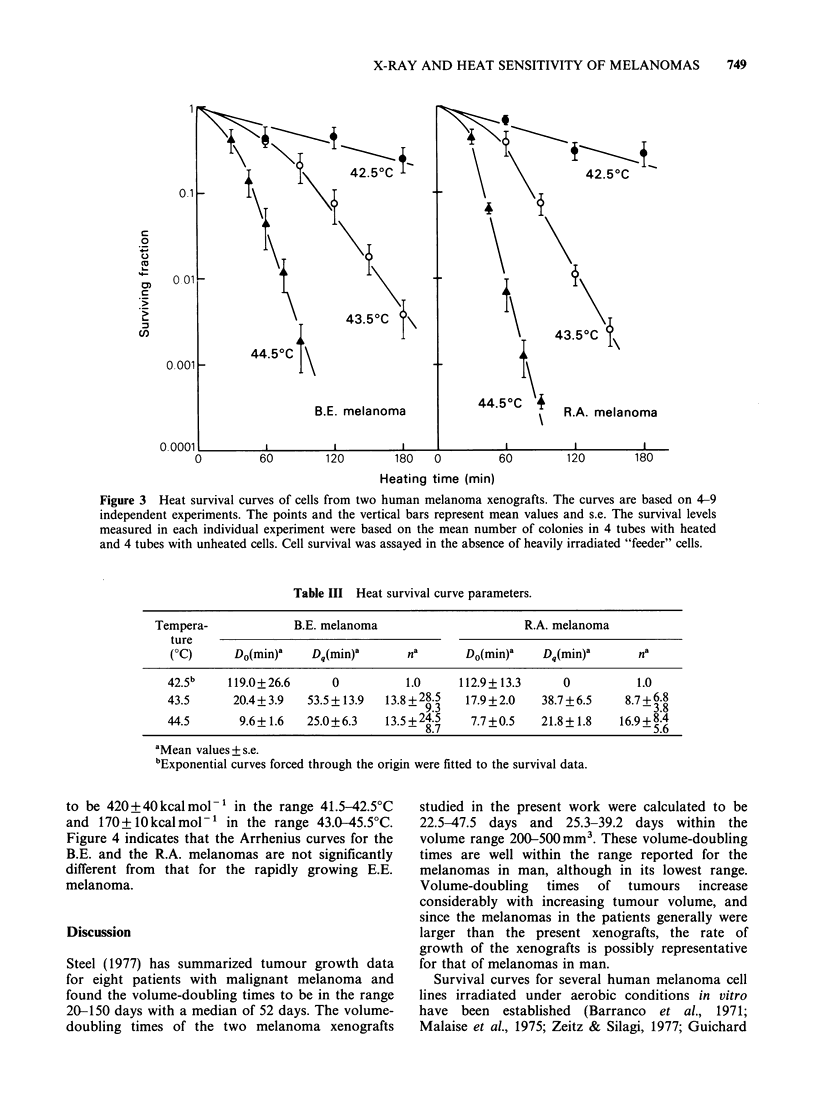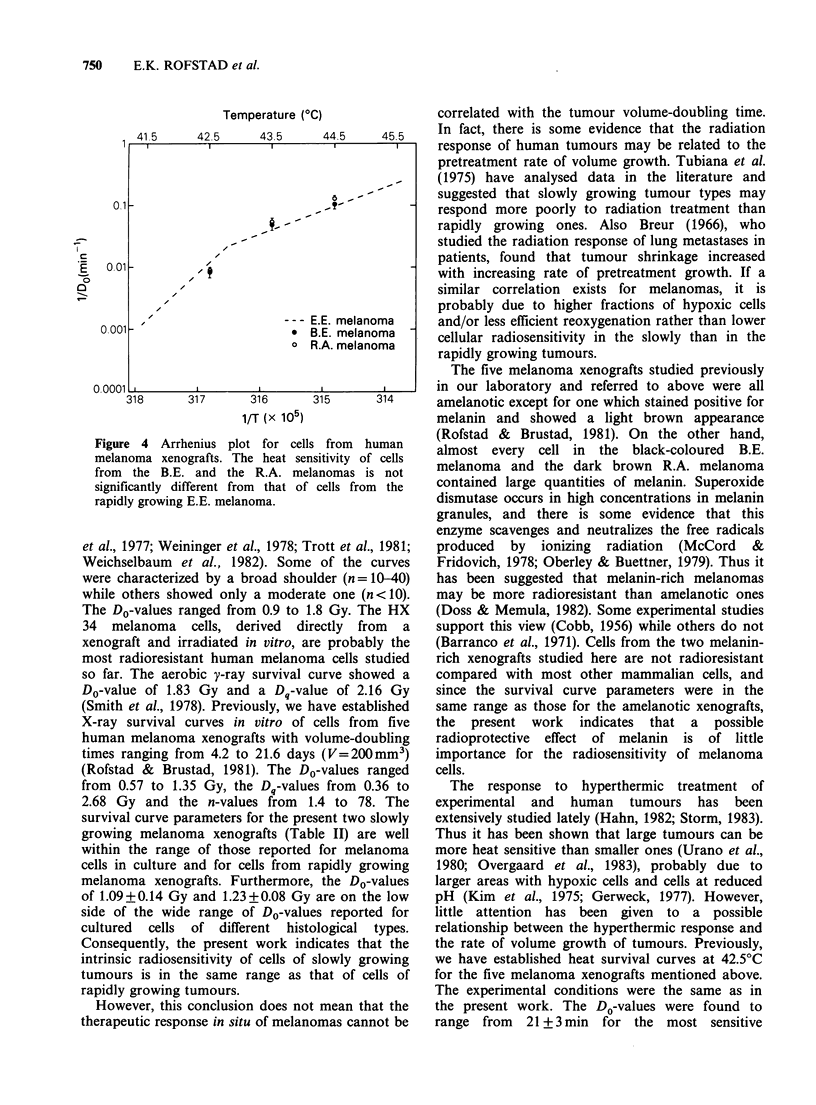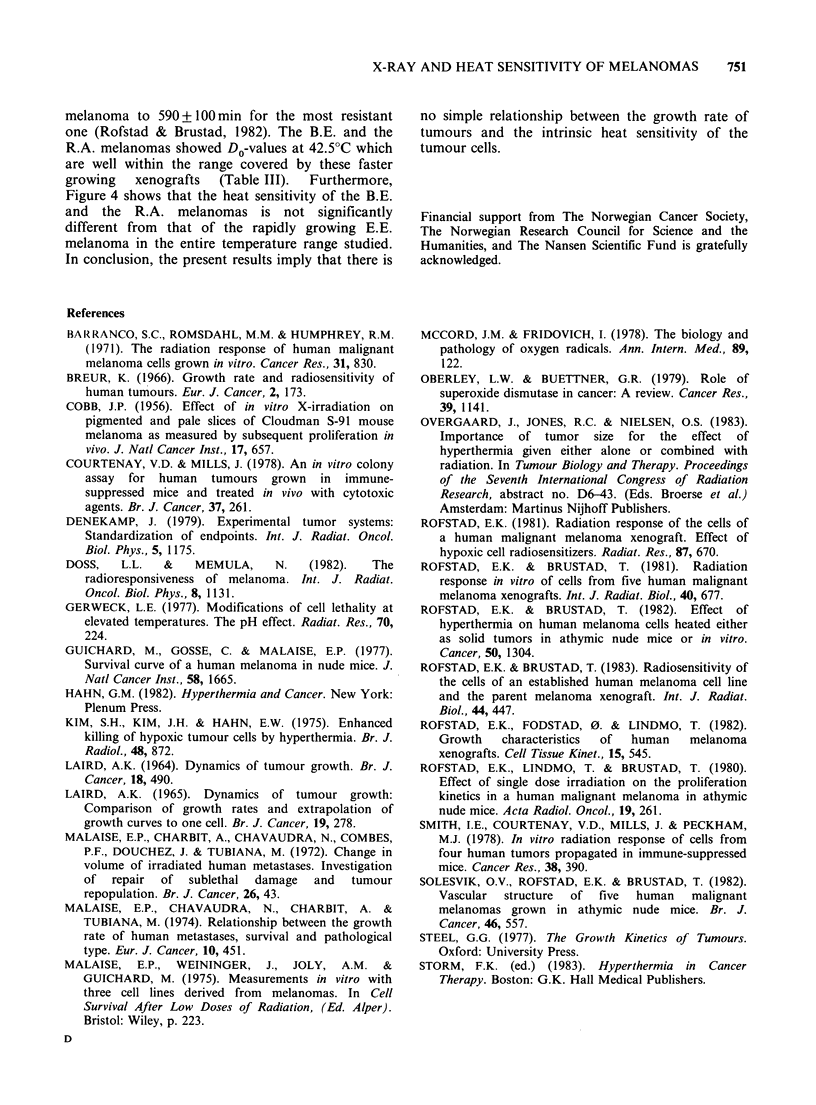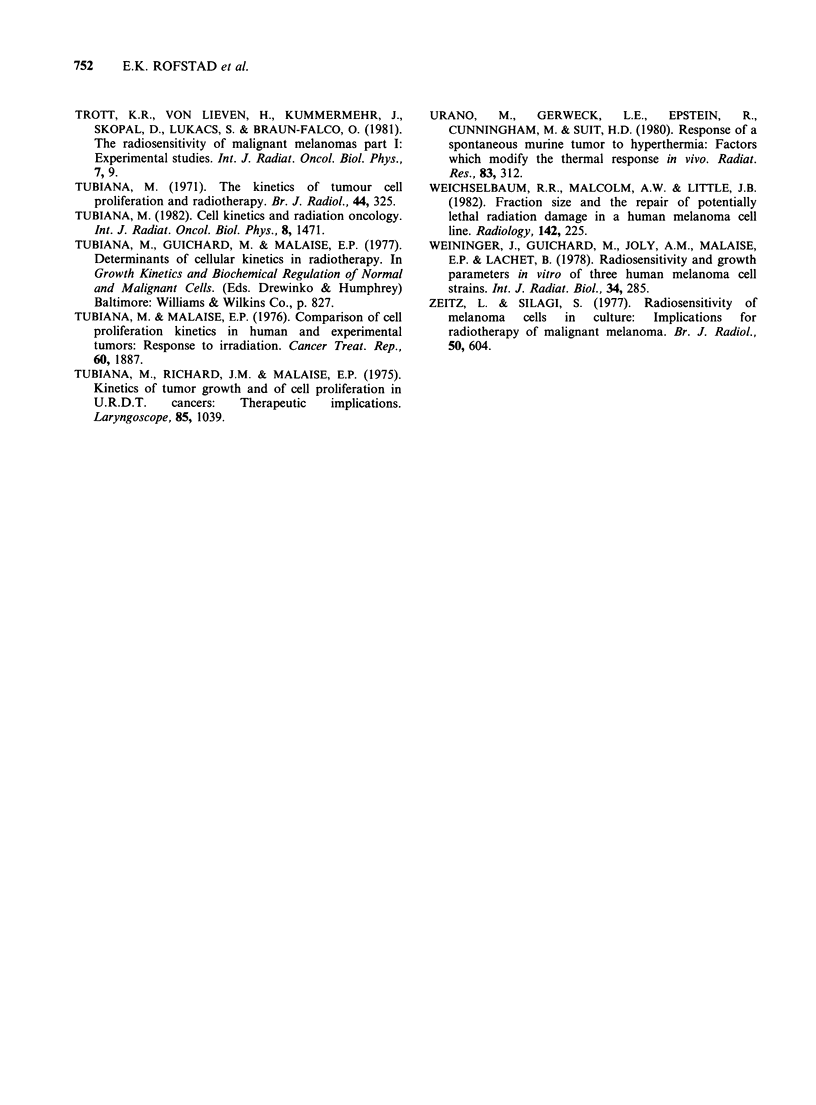# Radiation and heat sensitivity of cells from two slowly growing human melanoma xenografts.

**DOI:** 10.1038/bjc.1984.117

**Published:** 1984-06

**Authors:** E. K. Rofstad, A. Wahl, T. Brustad

## Abstract

The radiation and heat sensitivity of cells from two melanin-rich, slowly growing human melanoma xenografts (B.E. and R.A.) were studied. The volume-doubling times of the xenografts in the volume range 200-500 mm3 were found to be 22.5 47.5 days (B.E.) and 25.3-39.2 days (R.A.). The cells were suspended in culture medium during irradiation or heating, and the colony forming ability of the cells was assayed in soft agar. The X-ray survival curve parameters were found to be: Do = 1.09 +/- 0.14 Gy, Dq = 1.99 +/- 0.58 Gy (B.E.); Do = 1.23 +/- 0.08 Gy, Dq = 2.03 +/- 0.35 Gy (R.A.). The Do-values of the heat survival curves were found to be 119.0 +/- 26.6 min (42.5 degrees C), 20.4 +/- 3.9 min (43.5 degrees C) and 9.6 +/- 1.6 min (44.5 degrees C) for the B.E. melanoma and 112.9 +/- 13.3 min (42.5 degrees C), 17.9 +/- 2.0 min (43.5 degrees C) and 7.7 +/- 0.5 min (44.5 degrees C) for the R.A. melanoma. Both the radiation and the heat sensitivities of the cells are within the range of sensitivities reported for rapidly growing melanoma xenografts, suggesting that the intrinsic radiation and heat sensitivity of tumour cells are not strongly related to the rate of tumour growth prior to treatment.


*Br. J. Cancer* (1984), **49**, 745–752

### Radiation and heat sensitivity of cells from two slowly growing human melanoma xenografts

E.K. Rofstad, A. Wahl and T. Brustad

*Norsk Hydro's Institute for Cancer Research and The Norwegian Cancer Society, The Norwegian Radium Hospital, Montebello, Oslo 3, Norway*

**Summary** The radiation and heat sensitivity of cells from two melanin-rich, slowly growing human melanoma xenografts (B.E. and R.A.) were studied. The volume-doubling times of the xenografts in the volume range 200–500 mm$^3$ were found to be 22.5–47.5 days (B.E.) and 25.3–39.2 days (R.A.). The cells were suspended in culture medium during irradiation or heating, and the colony forming ability of the cells was assayed in soft agar. The X-ray survival curve parameters were found to be: $D_0 = 1.09 \pm 0.14$ Gy, $D_q = 1.99 \pm 0.58$ Gy (B.E.); $D_0 = 1.23 \pm 0.08$ Gy, $D_q = 2.03 \pm 0.35$ Gy (R.A.). The $D_0$-values of the heat survival curves were found to be $119.0 \pm 26.6$ min (42.5°C), $20.4 \pm 3.9$ min (43.5°C) and $9.6 \pm 1.6$ min (44.5°C) for the B.E. melanoma and $112.9 \pm 13.3$ min (42.5°C), $17.9 \pm 2.0$ min (43.5°C) and $7.7 \pm 0.5$ min (44.5°C) for the R.A. melanoma. Both the radiation and the heat sensitivities of the cells are within the range of sensitivities reported for rapidly growing melanoma xenografts, suggesting that the intrinsic radiation and heat sensitivity of tumour cells are not strongly related to the rate of tumour growth prior to treatment.

A wide variety of transplantable tumours of small rodents is currently used as models for human tumours in experimental cancer therapy research (Denekamp, 1979). Most of these tumours have volume-doubling times in the range 1–10 days (Steel, 1977), while the volume-doubling time of human tumours is usually in the range 10–200 days (Steel, 1977; Tubiana 1971; 1982). There is some evidence from clinical investigations that slowly growing tumours respond more poorly to radiation therapy and chemotherapy than do rapidly growing tumours (Breur, 1966; Malaise *et al.*, 1972; 1974; Tubiana & Malaise, 1976; Tubiana *et al.*, 1975; 1977). Conclusions drawn from therapy experiments with rapidly growing rodent tumours may therefore not be representative for human cancer. Consequently, there is a considerable need for experimental data obtained on slowly growing tumours.

Human tumour xenografts, grown in congenitally athymic mice or in immune-suppressed mice, usually show longer volume-doubling times than most transplantable rodent tumours. Previously, the response to radiation (Rofstad & Brustad, 1981) and hyperthermia (Rofstad & Brustad, 1982) of five human melanoma xenografts was studied in detail at our institute. The volume-doubling times of these tumours were in the range 4.2–21.6 days ($V = 200$ mm$^3$) (Rofstad *et al.*, 1982). Since these volume-doubling times are also shorter than those reported for most tumours in man, we have tried to establish other human melanoma xenografts with still slower growth. Two of the melanomas which were transplanted to athymic mice, may be of special interest in experimental cancer therapy for two reasons: Firstly, these melanomas have shown stable growth at a relatively low growth rate for several passages. Secondly, cells from the melanomas are able to form colonies when seeded in soft agar.

The purpose of the present work was to study the growth rate of these two melanoma xenografts as well as the intrinsic radiation and heat sensitivity of cells from the xenografts when treated under *in vitro* conditions. These radiation and heat sensitivities are discussed in relation to those of cells from rapidly growing melanoma xenografts and melanoma cells in culture.

### Materials and methods

### *Mice and tumours*

Female BALB/c/nu/nu/BOM mice, kept under specific pathogen-free (SPF) conditions, were used.

Two human melanomas (B.E. and R.A.) derived from metastases of patients at The Norwegian Radium Hospital were studied in the present work. The melanomas were transplanted into athymic mice without previous adaptation to *in vitro* culture conditions. The two parent metastases were similar histologically. Both cells and nuclei varied greatly in size and shape, and some mitoses were seen. The cells contained large quantities of melanin.

The tumours were grown serially in athymic mice implanting fragments, approximately

Correspondence: E.K. Rofstad.
Received 12 December 1983; accepted 21 February 1984.

© The Macmillan Press Ltd., 1984

$2\times2\times2$ mm in size, s.c. into recipient mice. Passages 17–19 (B.E.) and 8–12 (R.A.) were used in the present work. The tumours were carefully implanted at the same site in the flanks of the animals, and the tumour volumes were in the range 200–500 mm$^3$ when the radiation and the hyperthermia experiments were carried out. The B.E. and the R.A. melanoma xenografts showed a black and a dark brown appearance, respectively. Light- and electron-microscopic examinations showed the histology of the xenografts to be similar to that of the metastases from which they were derived, indicating that serial transplantation has not significantly changed the morphology of the melanomas.

*Growth*

Tumour growth can usually be described mathematically by a Compertz function (Laird, 1964; 1965):

$$V(t)=V(0)\exp\left[\tfrac{\alpha}{\beta}(1-e^{-\beta t})\right]$$

$$=V_{\max}\exp\left[-\tfrac{\alpha}{\beta}e^{-\beta t}\right]$$

where $V(t)$ is tumour volume at time $t$, $V(0)$ is initial tumour volume, $V_{\max}=\lim V(t)$ (usually referred to as the theoretical maximum tumour volume), and $\alpha$ and $\beta$ are constants. In the present study the parameters $\alpha$, $\beta$, $V_{\max}$ and the instantaneous tumour volume-doubling time ($T_d$) were calculated by Gompertz analysis as previously described for other melanoma xenografts (Rofstad *et al.*, 1982).

*Preparation of single cell suspensions*

Single cell suspensions were prepared from the tumours without the use of enzymes. The tumours were finely minced in culture medium (Ham's F12 medium with 20% foetal calf serum, 250 mg l$^{-1}$ penicillin and 50 mg l$^{-1}$ streptomycin (Gibco-Biocult)) by means of a scalpel and a pair of tweezers. The tissue suspensions were filtered through 30 $\mu$m filters (Nytal, Schweizerische Seidengazefabrik AG). The cell concentration was determined by the use of a haemocytometer viewed through a microscope with phase contrast optics. Cells having an intact and smooth outline with a bright halo were counted as viable. The cell suspensions were diluted to appropriate concentrations in culture medium.

*Irradiation*

A Siemens "Stabilipan" X-ray unit, operated at 220 kV, 19–20 mA and with 0.5 mm Cu filtration, was used for irradiation. The cell suspensions, which were irradiated under aerobic conditions at a dose-rate of 3.0 Gy min$^{-1}$, were kept in glass Carrel flasks during exposure. The irradiation was performed at room temperature.

*Heating*

Single cell suspensions were kept during heating in glass tubes with ground glass stoppers. Stopcock grease was applied in the joints to make the tubes gas-tight. Immediately before heating, the tubes were flushed (5% $CO_2$ in air) and carefully sealed. The pH of the cell suspensions was 7.4. The tubes were immersed in a thermostatically regulated water-bath during the heat exposure. The cell suspensions reached the temperature in the water-bath within 4 min. The tubes were agitated during the heating to keep the cells suspended.

*In vitro colony assay*

The colony forming ability of the cells was measured by using the soft agar colony assay developed by Courtenay & Mills (1978). The soft agar was prepared from powdered agar (Bacto agar, Difco), suspensions of melanoma cells, and erythrocytes from August rats according to the procedure described previously (Rofstad, 1981). The plating efficiency was significantly enhanced in the presence of erythrocytes, probably because a growth factor was released from the cells (Courtenay & Mills, 1978).

Aliquots of 1 ml of soft agar with the appropriate concentration of melanoma cells were seeded in plastic tubes (Falcon 2057 tubes). The cells were incubated at 37°C for 25–32 days in an atmosphere of 5% $O_2$, 5% $CO_2$ and 90% $N_2$. At that time the colonies had reached a mean diameter of $\sim 250\,\mu$m, indicating that the cells proliferate slowly in the soft agar. Culture medium (2 ml) was added 5 days after seeding and changed weekly. Colonies were counted by the use of a stereomicroscope. Cells giving rise to colonies $>50$ cells were scored as surviving. The plating efficiency of cells from both melanomas was in the range 1–5%. The effect of "feeder" cells on cell survival was measured by adding $10^5$ heavily irradiated cells (100 Gy) to each tube. Cells from the same tumours as those used in the survival experiments were used as "feeder" cells. Details of the experimental procedure are reported elsewhere (Rofstad, 1981).

### Results

The volumetric growth data for both xenografts were well fitted by Gompertz curves (Figure 1). The Gompertz parameters are presented in Table I. The volume-doubling times in the volume range 200–

**Figure 1** Gompertz growth curves for two human melanoma xenografts. The curves are based on the mean volume of 16 B.E. (●) and 13 R.A. (○) individual tumours from one passage representative for each xenograft. Vertical bars = s.e.

**Table I** Gompertz growth curve parameters.

| Parameter[a] | B.E. melanoma | R.A. melanoma |
|---|---|---|
| $\alpha$(day$^{-1}$) | 0.136 ± 0.002 | 0.077 ± 0.001 |
| $\beta$(day$^{-1}$) | 0.0172 ± 0.0002 | 0.0105 ± 0.0002 |
| $V_{max}$(mm$^3$) | 1730 ± 45 | 3900 ± 150 |
| $T_d$(days) ($V$ = 200 mm$^3$) | 22.5 | 25.3 |
| $T_d$(days) ($V$ = 500 mm$^3$) | 47.5 | 39.2 |

[a]Mean values ± s.e.

500 mm$^3$ were 22.5–47.5 days for the B.E. melanoma and 25.3–39.2 days for the R.A. melanoma (Table I).

Growth experiments *in vitro* showed that addition of heavily irradiated "feeder" cells did not influence the surviving fractions measured for the B.E. melanoma, either the cells were exposed to radiation or heat. Thus, "feeder" cells were not used routinely in the survival experiments with this melanoma. On the other hand, the surviving fractions measured for the R.A. melanoma after exposure to X-rays were lower in the presence than in the absence of "feeder" cells (Figure 2), mainly because of higher plating efficiency when "feeder" cells were present. Complete survival curves with and without the use of "feeder" cells were therefore established for cells from the R.A. melanoma also when heated at 44.5°C. The number of colonies was always higher in the presence of heavily irradiated "feeder" cells but the survival levels were not significantly different. This observation indicates that in experiments where "feeder" cells were not used, cells inactivated by heat did not stimulate colony formation as did cells inactivated by radiation. Thus, heat survival curves at 42.5 and 43.5°C were established in the absence of "feeder" cells only. The large differences between the surviving fractions measured in the presence and the absence of heavily irradiated cells for the R.A. melanoma exposed to X-rays (Figure 2) demonstrate the necessity of investigating the effects of "feeder" cells when colony forming ability of cells from human tumour xenografts is studied by the soft agar technique.

X-ray survival curves for the two melanoma xenografts are presented in Figure 2. Exponential curves were fitted to the data in the dose range 4.0–12.0 Gy. The $D_0$-values were found to be 1.09 ± 0.14 Gy (B.E.) and 1.23 ± 0.08 Gy (R.A.). The parameters of the survival curves are summarized in Table II.

**Figure 2** X-ray survival curves of cells from two human melanoma xenografts. The curves are based on 3–5 independent experiments. The points and the vertical bars represent mean values and s.e. The survival levels measured in each individual experiment were based on the mean number of colonies in 4 tubes with irradiated and 4 tubes with unirradiated cells. Cell survival was assayed in the absence (●, ▲) or in the presence (○) of heavily irradiated "feeder" cells.

**Table II** X-ray survival curve parameters.

| Parameter[a] | B.E. melanoma | R.A.[b] melanoma |
|---|---|---|
| $D_0$(Gy) | $1.09 \pm 0.14$ | $1.23 \pm 0.08$ |
| $D_q$(Gy) | $1.99 \pm 0.58$ | $2.03 \pm 0.35$ |
| $n$ | $6.2 \pm ^{7.1}_{3.3}$ | $5.2 \pm ^{2.4}_{1.7}$ |

[a]Mean values ± s.e.

[b]Heavily irradiated "feeder" cells ($10^5$ cells per tube) were added.

Figure 3 shows heat survival curves for the B.E. and the R.A. melanomas. The data at 43.5 and 44.5°C are clearly consistent with survival curves exhibiting an initial shoulder, while the cell survival at 42.5°C does not appear to deviate from exponential kinetics. Exponential curves were fitted to the data by the method of least squares. For the R.A. melanoma all data were included in the regression analysis, while for the B.E. melanoma the data at 30 min (44.5°C) and 60 min (43.5°C) were found to be in the shoulder region and were not included in the analysis. The criteria used to define the shoulder and the exponential regions of the curves have been described in detail previously (Rofstad & Brustad, 1983). Survival curve parameters after heat treatment are presented in Table III.

The heat inactivation data for the B.E. and the R.A. melanomas are presented in an Arrhenius plot in Figure 4 together with the Arrhenius curve for the E.E. melanoma. The E.E. melanoma is a human tumour xenograft which has been maintained in athymic mice for several years by using the same transplantation technique as that described for the B.E. and the R.A. melanomas. The origin and some biological characteristics of this xenograft have been reported previously (Rofstad *et al.*, 1980; 1982; Solesvik *et al.*, 1982). The E.E. melanoma is a rapidly growing tumour with a volume-doubling time of 4.4 days at $V = 200\,mm^3$. The Arrhenius curve for this melanoma was determined from 18 heat survival curves in the temperature range 41.5–45.5°C (Rofstad & Brustad, manuscript in preparation) and is presented without experimental points in Figure 4. The activation energy was found

**Figure 3** Heat survival curves of cells from two human melanoma xenografts. The curves are based on 4–9 independent experiments. The points and the vertical bars represent mean values and s.e. The survival levels measured in each individual experiment were based on the mean number of colonies in 4 tubes with heated and 4 tubes with unheated cells. Cell survival was assayed in the absence of heavily irradiated "feeder" cells.

**Table III** Heat survival curve parameters.

| Temperature (°C) | B.E. melanoma | | | R.A. melanoma | | |
|---|---|---|---|---|---|---|
| | $D_0$(min)[a] | $D_q$(min)[a] | $n$[a] | $D_0$(min)[a] | $D_q$(min)[a] | $n$[a] |
| 42.5[b] | 119.0±26.6 | 0 | 1.0 | 112.9±13.3 | 0 | 1.0 |
| 43.5 | 20.4±3.9 | 53.5±13.9 | $13.8\pm^{28.5}_{9.3}$ | 17.9±2.0 | 38.7±6.5 | $8.7\pm^{6.8}_{3.8}$ |
| 44.5 | 9.6±1.6 | 25.0±6.3 | $13.5\pm^{24.5}_{8.7}$ | 7.7±0.5 | 21.8±1.8 | $16.9\pm^{8.4}_{5.6}$ |

[a]Mean values±s.e.
[b]Exponential curves forced through the origin were fitted to the survival data.

to be 420±40 kcal mol$^{-1}$ in the range 41.5–42.5°C and 170±10 kcal mol$^{-1}$ in the range 43.0–45.5°C. Figure 4 indicates that the Arrhenius curves for the B.E. and the R.A. melanomas are not significantly different from that for the rapidly growing E.E. melanoma.

### Discussion

Steel (1977) has summarized tumour growth data for eight patients with malignant melanoma and found the volume-doubling times to be in the range 20–150 days with a median of 52 days. The volume-doubling times of the two melanoma xenografts studied in the present work were calculated to be 22.5–47.5 days and 25.3–39.2 days within the volume range 200–500 mm$^3$. These volume-doubling times are well within the range reported for the melanomas in man, although in its lowest range. Volume-doubling times of tumours increase considerably with increasing tumour volume, and since the melanomas in the patients generally were larger than the present xenografts, the rate of growth of the xenografts is possibly representative for that of melanomas in man.

Survival curves for several human melanoma cell lines irradiated under aerobic conditions *in vitro* have been established (Barranco *et al.*, 1971; Malaise *et al.*, 1975; Zeitz & Silagi, 1977; Guichard

**Figure 4** Arrhenius plot for cells from human melanoma xenografts. The heat sensitivity of cells from the B.E. and the R.A. melanomas is not significantly different from that of cells from the rapidly growing E.E. melanoma.

*et al.*, 1977; Weininger *et al.*, 1978; Trott *et al.*, 1981; Weichselbaum *et al.*, 1982). Some of the curves were characterized by a broad shoulder ($n = 10$–$40$) while others showed only a moderate one ($n < 10$). The $D_0$-values ranged from 0.9 to 1.8 Gy. The HX 34 melanoma cells, derived directly from a xenograft and irradiated *in vitro*, are probably the most radioresistant human melanoma cells studied so far. The aerobic $\gamma$-ray survival curve showed a $D_0$-value of 1.83 Gy and a $D_q$-value of 2.16 Gy (Smith *et al.*, 1978). Previously, we have established X-ray survival curves *in vitro* of cells from five human melanoma xenografts with volume-doubling times ranging from 4.2 to 21.6 days ($V = 200\,mm^3$) (Rofstad & Brustad, 1981). The $D_0$-values ranged from 0.57 to 1.35 Gy, the $D_q$-values from 0.36 to 2.68 Gy and the $n$-values from 1.4 to 78. The survival curve parameters for the present two slowly growing melanoma xenografts (Table II) are well within the range of those reported for melanoma cells in culture and for cells from rapidly growing melanoma xenografts. Furthermore, the $D_0$-values of $1.09 \pm 0.14$ Gy and $1.23 \pm 0.08$ Gy are on the low side of the wide range of $D_0$-values reported for cultured cells of different histological types. Consequently, the present work indicates that the intrinsic radiosensitivity of cells of slowly growing tumours is in the same range as that of cells of rapidly growing tumours.

However, this conclusion does not mean that the therapeutic response *in situ* of melanomas cannot be correlated with the tumour volume-doubling time. In fact, there is some evidence that the radiation response of human tumours may be related to the pretreatment rate of volume growth. Tubiana *et al.* (1975) have analysed data in the literature and suggested that slowly growing tumour types may respond more poorly to radiation treatment than rapidly growing ones. Also Breur (1966), who studied the radiation response of lung metastases in patients, found that tumour shrinkage increased with increasing rate of pretreatment growth. If a similar correlation exists for melanomas, it is probably due to higher fractions of hypoxic cells and/or less efficient reoxygenation rather than lower cellular radiosensitivity in the slowly than in the rapidly growing tumours.

The five melanoma xenografts studied previously in our laboratory and referred to above were all amelanotic except for one which stained positive for melanin and showed a light brown appearance (Rofstad & Brustad, 1981). On the other hand, almost every cell in the black-coloured B.E. melanoma and the dark brown R.A. melanoma contained large quantities of melanin. Superoxide dismutase occurs in high concentrations in melanin granules, and there is some evidence that this enzyme scavenges and neutralizes the free radicals produced by ionizing radiation (McCord & Fridovich, 1978; Oberley & Buettner, 1979). Thus it has been suggested that melanin-rich melanomas may be more radioresistant than amelanotic ones (Doss & Memula, 1982). Some experimental studies support this view (Cobb, 1956) while others do not (Barranco *et al.*, 1971). Cells from the two melanin-rich xenografts studied here are not radioresistant compared with most other mammalian cells, and since the survival curve parameters were in the same range as those for the amelanotic xenografts, the present work indicates that a possible radioprotective effect of melanin is of little importance for the radiosensitivity of melanoma cells.

The response to hyperthermic treatment of experimental and human tumours has been extensively studied lately (Hahn, 1982; Storm, 1983). Thus it has been shown that large tumours can be more heat sensitive than smaller ones (Urano *et al.*, 1980; Overgaard *et al.*, 1983), probably due to larger areas with hypoxic cells and cells at reduced pH (Kim *et al.*, 1975; Gerweck, 1977). However, little attention has been given to a possible relationship between the hyperthermic response and the rate of volume growth of tumours. Previously, we have established heat survival curves at 42.5°C for the five melanoma xenografts mentioned above. The experimental conditions were the same as in the present work. The $D_0$-values were found to range from $21 \pm 3$ min for the most sensitive melanoma to 590 ± 100 min for the most resistant one (Rofstad & Brustad, 1982). The B.E. and the R.A. melanomas showed $D_0$-values at 42.5°C which are well within the range covered by these faster growing xenografts (Table III). Furthermore, Figure 4 shows that the heat sensitivity of the B.E. and the R.A. melanomas is not significantly different from that of the rapidly growing E.E. melanoma in the entire temperature range studied. In conclusion, the present results imply that there is no simple relationship between the growth rate of tumours and the intrinsic heat sensitivity of the tumour cells.

Financial support from The Norwegian Cancer Society, The Norwegian Research Council for Science and the Humanities, and The Nansen Scientific Fund is gratefully acknowledged.

### References

BARRANCO, S.C., ROMSDAHL, M.M. & HUMPHREY, R.M. (1971). The radiation response of human malignant melanoma cells grown *in vitro*. *Cancer Res.*, **31**, 830.

BREUR, K. (1966). Growth rate and radiosensitivity of human tumours. *Eur. J. Cancer*, **2**, 173.

COBB, J.P. (1956). Effect of *in vitro* X-irradiation on pigmented and pale slices of Cloudman S-91 mouse melanoma as measured by subsequent proliferation *in vivo*. *J. Natl Cancer Inst.*, **17**, 657.

COURTENAY, V.D. & MILLS, J. (1978). An *in vitro* colony assay for human tumours grown in immune-suppressed mice and treated *in vivo* with cytotoxic agents. *Br. J. Cancer*, **37**, 261.

DENEKAMP, J. (1979). Experimental tumor systems: Standardization of endpoints. *Int. J. Radiat. Oncol. Biol. Phys.*, **5**, 1175.

DOSS, L.L. & MEMULA, N. (1982). The radioresponsiveness of melanoma. *Int. J. Radiat. Oncol. Biol. Phys.*, **8**, 1131.

GERWECK, L.E. (1977). Modifications of cell lethality at elevated temperatures. The pH effect. *Radiat. Res.*, **70**, 224.

GUICHARD, M., GOSSE, C. & MALAISE, E.P. (1977). Survival curve of a human melanoma in nude mice. *J. Natl Cancer Inst.*, **58**, 1665.

HAHN, G.M. (1982). *Hyperthermia and Cancer*. New York: Plenum Press.

KIM, S.H., KIM, J.H. & HAHN, E.W. (1975). Enhanced killing of hypoxic tumour cells by hyperthermia. *Br. J. Radiol.*, **48**, 872.

LAIRD, A.K. (1964). Dynamics of tumour growth. *Br. J. Cancer*, **18**, 490.

LAIRD, A.K. (1965). Dynamics of tumour growth: Comparison of growth rates and extrapolation of growth curves to one cell. *Br. J. Cancer*, **19**, 278.

MALAISE, E.P., CHARBIT, A., CHAVAUDRA, N., COMBES, P.F., DOUCHEZ, J. & TUBIANA, M. (1972). Change in volume of irradiated human metastases. Investigation of repair of sublethal damage and tumour repopulation. *Br. J. Cancer*, **26**, 43.

MALAISE, E.P., CHAVAUDRA, N., CHARBIT, A. & TUBIANA, M. (1974). Relationship between the growth rate of human metastases, survival and pathological type. *Eur. J. Cancer*, **10**, 451.

MALAISE, E.P., WEININGER, J., JOLY, A.M. & GUICHARD, M. (1975). Measurements *in vitro* with three cell lines derived from melanomas. In *Cell Survival After Low Doses of Radiation, (Ed. Alper)*. Bristol: Wiley, p. 223.

MCCORD, J.M. & FRIDOVICH, I. (1978). The biology and pathology of oxygen radicals. *Ann. Intern. Med.*, **89**, 122.

OBERLEY, L.W. & BUETTNER, G.R. (1979). Role of superoxide dismutase in cancer: A review. *Cancer Res.*, **39**, 1141.

OVERGAARD, J., JONES, R.C. & NIELSEN, O.S. (1983). Importance of tumor size for the effect of hyperthermia given either alone or combined with radiation. In *Tumour Biology and Therapy. Proceedings of the Seventh International Congress of Radiation Research*, abstract no. D6–43. (Eds. Broerse *et al.*) Amsterdam: Martinus Nijhoff Publishers.

ROFSTAD, E.K. (1981). Radiation response of the cells of a human malignant melanoma xenograft. Effect of hypoxic cell radiosensitizers. *Radiat. Res.*, **87**, 670.

ROFSTAD, E.K. & BRUSTAD, T. (1981). Radiation response *in vitro* of cells from five human malignant melanoma xenografts. *Int. J. Radiat. Biol.*, **40**, 677.

ROFSTAD, E.K. & BRUSTAD, T. (1982). Effect of hyperthermia on human melanoma cells heated either as solid tumors in athymic nude mice or *in vitro*. *Cancer*, **50**, 1304.

ROFSTAD, E.K. & BRUSTAD, T. (1983). Radiosensitivity of the cells of an established human melanoma cell line and the parent melanoma xenograft. *Int. J. Radiat. Biol.*, **44**, 447.

ROFSTAD, E.K., FODSTAD, Ø. & LINDMO, T. (1982). Growth characteristics of human melanoma xenografts. *Cell Tissue Kinet.*, **15**, 545.

ROFSTAD, E.K., LINDMO, T. & BRUSTAD, T. (1980). Effect of single dose irradiation on the proliferation kinetics in a human malignant melanoma in athymic nude mice. *Acta Radiol. Oncol.*, **19**, 261.

SMITH, I.E., COURTENAY, V.D., MILLS, J. & PECKHAM, M.J. (1978). *In vitro* radiation response of cells from four human tumors propagated in immune-suppressed mice. *Cancer Res.*, **38**, 390.

SOLESVIK, O.V., ROFSTAD, E.K. & BRUSTAD, T. (1982). Vascular structure of five human malignant melanomas grown in athymic nude mice. *Br. J. Cancer*, **46**, 557.

STEEL, G.G. (1977). *The Growth Kinetics of Tumours*. Oxford: University Press.

STORM, F.K. (ed.) (1983). *Hyperthermia in Cancer Therapy*. Boston: G.K. Hall Medical Publishers.

TROTT, K.R., VON LIEVEN, H., KUMMERMEHR, J., SKOPAL, D., LUKACS, S. & BRAUN-FALCO, O. (1981). The radiosensitivity of malignant melanomas part I: Experimental studies. *Int. J. Radiat. Oncol. Biol. Phys.*, **7**, 9.

TUBIANA, M. (1971). The kinetics of tumour cell proliferation and radiotherapy. *Br. J. Radiol.*, **44**, 325.

TUBIANA, M. (1982). Cell kinetics and radiation oncology. *Int. J. Radiat. Oncol. Biol. Phys.*, **8**, 1471.

TUBIANA, M., GUICHARD, M. & MALAISE, E.P. (1977). Determinants of cellular kinetics in radiotherapy. In *Growth Kinetics and Biochemical Regulation of Normal and Malignant Cells*. (Eds. Drewinko & Humphrey) Baltimore: Williams & Wilkins Co., p. 827.

TUBIANA, M. & MALAISE, E.P. (1976). Comparison of cell proliferation kinetics in human and experimental tumors: Response to irradiation. *Cancer Treat. Rep.*, **60**, 1887.

TUBIANA, M., RICHARD, J.M. & MALAISE, E.P. (1975). Kinetics of tumor growth and of cell proliferation in U.R.D.T. cancers: Therapeutic implications. *Laryngoscope*, **85**, 1039.

URANO, M., GERWECK, L.E., EPSTEIN, R., CUNNINGHAM, M. & SUIT, H.D. (1980). Response of a spontaneous murine tumor to hyperthermia: Factors which modify the thermal response *in vivo*. *Radiat. Res.*, **83**, 312.

WEICHSELBAUM, R.R., MALCOLM, A.W. & LITTLE, J.B. (1982). Fraction size and the repair of potentially lethal radiation damage in a human melanoma cell line. *Radiology*, **142**, 225.

WEININGER, J., GUICHARD, M., JOLY, A.M., MALAISE, E.P. & LACHET, B. (1978). Radiosensitivity and growth parameters *in vitro* of three human melanoma cell strains. *Int. J. Radiat. Biol.*, **34**, 285.

ZEITZ, L. & SILAGI, S. (1977). Radiosensitivity of melanoma cells in culture: Implications for radiotherapy of malignant melanoma. *Br. J. Radiol.*, **50**, 604.